# Early detection of gastric cancer in China: progress and opportunities

**DOI:** 10.20892/j.issn.2095-3941.2022.0655

**Published:** 2022-12-12

**Authors:** Hengmin Xu, Wenqing Li

**Affiliations:** 1 Key Laboratory of Carcinogenesis and Translational Research (Ministry of Education/Beijing), Department of Cancer Epidemiology, Peking University Cancer Hospital and Institute, Beijing 100142, China

In the past decade, the Chinese government has committed to promoting gastric cancer (GC) prevention and control^1,2^. GC is a major malignancy globally, and nearly half of GC cases and specific deaths worldwide occur in China; moreover, GC is frequently diagnosed in local advanced or advanced stages with poor prognosis^3–5^. GC progression is known to involve a multi-stage evolutionary cascade from precancerous gastric lesions, thus theoretically enabling the identification of high-risk populations and early detection of GC^6^. However, practically accomplishing this goal remains challenging for most cases in real-world settings^7^.

GC screening is aimed at early detection and early diagnosis (“secondary prevention”), which currently relies primarily on endoscopic examinations and is widely accepted as the mainstream of current GC prevention efforts. Electronic endoscopy with gastric histopathological diagnosis is recognized as the gold standard for GC screening programs worldwide. Although population coverage still requires improvement, the effectiveness of GC screening programs in China had already been demonstrated. On the basis of prospective follow-up of screened *vs.* unscreened individuals, beneficial effects of endoscopic screening on decreasing GC incidence and mortality have been reported^2,8^. Nonetheless, endoscopic screening for the entire population is unrealistic in China, owing to the understaffing of well-trained endoscopists and pathologists, deficiency in endoscopic facilities, disparities in local socioeconomic status, and both direct and indirect expenses. Individuals may avoid endoscopic examination because of its invasiveness, related epigastric discomfort, and concerns regarding other rare but intolerable safety issues. In an era of precision medicine, refined risk prediction and the development of complementary biomarkers with optimal performance are urgently needed^9^. Robust evaluations are required to provide high-quality evidence in appropriate populations to achieve personalized screening strategies. Here, we describe the historical milestones in GC screening programs in China and summarize evidence of the effectiveness of screening in GC prevention, including from a health economic perspective. We also comment on the opportunities for overcoming major challenges and elucidating key elements to advance individualized GC screening and prevention.

## National GC screening programs in China

**Figure 1:**
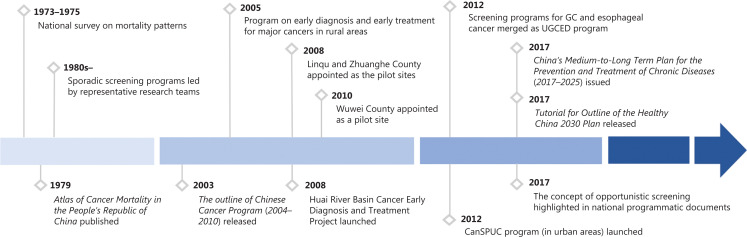
Key milestones for GC screening in China. UGCED, Upper Gastrointestinal Cancer Early Detection. CanSPUC, Cancer Screening Program in Urban China.

Unlike Japan and Korea, which have established nationwide programs^7^, GC screening in China has targeted selected high-risk areas (**Figure 1**).

### Screening efforts in the 20^th^ century

In 1973–1975, a national survey investigated the mortality patterns, particularly for cancers, in China, in accordance with the directive from late premier Zhou En-lai to track the occurrence and distribution of cancers. On the basis of this survey, *Atlas of Cancer Mortality in the People’s Republic of China*, published in 1979, showed geographical aggregation of major malignant tumors, thus providing a strong basis for subsequent research in high-risk areas for GC, notably Linqu (Shandong province), Zhuanghe (Liaoning province), Wuwei (Gansu province), and Changle (Fujian province). Although nationwide screening for GC was not yet initiated, epidemiologic studies have been conducted on the prevalence, incidence, and risk factors of GC in high-risk areas. For example, endoscopic screening in 1989–1990 involving 3,433 individuals in Linqu revealed a pervasive presence of precancerous gastric lesions^10,11^ and supported the stepwise progression of precancerous gastric lesions to GC, on the basis of prospective follow-up of study participants^6,12^. During a 10-year follow-up of this cohort, several repeated endoscopic examinations indicated a GC early detection rate (50%–67%) and 5-year survival rate (63.7%) much higher than the general levels reported in hospital-based studies in China^13^. In Zhuanghe, two-stage GC screening integrating serum pepsinogen detection and subsequent endoscopic examination was performed among 7,036 participants from 1996 to 2005, and a GC early detection rate of 56.8%–64% was achieved^14^.

### Population-based nationwide GC screening programs in the 2000s

*The Outline of Chinese Cancer Program (2004–2010)* released by the former Ministry of Health (MoH) of China proclaimed the inception of government-funded nationwide cancer prevention programs. In 2005, the Disease Prevention and Control Division of the former MoH and the China Cancer Foundation officially heralded the program on early diagnosis and treatment for major cancers in rural areas, supported by the Central Budget Funding Project of the Ministry of Finance. Linqu County in Shandong province and Zhuanghe County in Liaoning province, 2 known high-risk rural areas in China, were selected as the pilot sites for a GC screening program. In 2008, 3,133 volunteers in these 2 areas received endoscopic examinations, thus marking the inception of the first government-funded GC screening program in China. The Huai River Basin Cancer Early Diagnosis and Treatment Project was also launched in the same year. In 2010, Wuwei County in Gansu province was added as a new screening site. In 2012, the screening programs for GC and esophageal cancer were merged to create the national Upper Gastrointestinal Cancer Early Detection (UGCED) program, wherein residents 40–69 years of age in selected high-risk rural areas were eligible for early diagnosis of GC and esophageal cancer free of charge. Individuals with previous diagnoses of cancer (except nonmelanoma skin cancer), bleeding disorder, heart failure, renal disease, liver disease, emphysema, or other life-threatening illnesses were excluded. As of 2021, more than 2.6 million eligible rural inhabitants had received gastroendoscopy, and more than 70% of upper gastrointestinal (UGI) cancers were detected in early stages^15^.

A population-based nationwide GC screening program for urban inhabitants was not available until 2012, when the Cancer Screening Program in Urban China (CanSPUC) was initiated jointly by the Ministry of Finance and the former MoH, with a focus on screening for lung cancer, breast cancer, UGI cancer, colorectal cancer, and liver cancer. As of 2018, 143,000 participants had received UGI endoscopic examination in the CanSPUC program^16^.

## Effectiveness of GC screening

### Beneficial effects on GC incidence and mortality

Endoscopic examinations directly lead to early detection and early diagnosis, and thus favorable prognosis of GC. During a 10-year follow-up of 637,500 participants 40–69 years of age from 6 high-risk areas for esophageal cancer in China, Chen et al.^8^ have found that endoscopic examination screening significantly decreased the incidence of non-cardia invasive GC [relative risk (RR) = 0.66, 95% CI: 0.59–0.73] and mortality from non-cardia GC (RR = 0.38, 95% CI: 0.33–0.45) and cardia invasive GC (RR = 0.58, 95% CI: 0.49–0.68). Recently, our team conducted a prospective study (2012–2018) in 375,800 individuals from the high-risk Linqu area in Shandong province, China, which indicated that endoscopic screening significantly decreased the incidence of invasive GC (RR = 0.69, 95% CI: 0.52–0.92) and mortality (RR = 0.33, 95% CI: 0.20–0.56), particularly for non-cardia GC. Repeated endoscopy further strengthened the decrease in GC-specific death, with 5-year survival rates of 31.9%, 73.4%, and 90.2% for GCs in the unscreened group, the group screened once, and the group with repeated screening, respectively^2^. These population-based studies support that endoscopic screening prevents the incidence and mortality of invasive GC, owing to the detection of precancerous lesions, and additionally improves GC prognosis. The effectiveness of these measures is enhanced by repeated screening^2^, thus further corroborating the public health implications of expanding GC screening and early detection.

### Evidence of cost-effectiveness

Given the limited resources of the health care system, balancing the costs and effects of GC screening is key to informing future government actions and policy making. Xia et al.^17^ have reported marked increases in the incremental cost-effectiveness ratio ranging between US $1343 and $3035 per quality-adjusted life-year for several screening strategies with different starting ages and frequencies, compared with no screening over a lifetime, thereby supporting the cost-effectiveness of endoscopic screening for UGI cancers in high-risk areas. Compared with the strategy of nationwide general screening, a decision analysis has further predicted that targeted screening results in 44%–49% lower costs per quality-adjusted life-year gained over daily routine care, thus suggesting the favorable cost-effectiveness of screening in a personalized approach for high-risk population subgroups^18^. Indeed, even among high-risk populations, the screening frequency must be adjusted to personal risk so that endoscopy-associated resources can be saved^18^.

## Opportunities for improving early GC detection in China

### Integrating biomarkers for GC screening tailored to personalized risk

Because a targeted GC screening strategy is an optimized approach, using biomarkers to assess risk would support risk stratification and the identification of individuals more likely to develop GC. Blood-based biomarkers have been an area of great promise, because the blood may indicate overall health status and capture early molecular signatures in a non-invasive approach, which may also facilitate the dynamic monitoring of biomarkers. Blood pepsinogen (PG), gastrin-17 (G-17), and *H. pylori* antibodies have long been examined for screening and early detection of GC, and continued efforts have examined the combination of PG with other factors (**Table 1**). Recent advances in bioinformatics and genetic testing have accelerated research on liquid biopsies, and changes in circulating tumor cells (CTCs) or cell-free DNA (cfDNA) have been considered potentially suitable markers for GC screening. Numerous studies have studied CTCs for the prognosis of GC, owing to their ability to be dynamically monitored^24^, but debate persists regarding the use of CTCs in early diagnosis of GC. A meta-analysis has indicated a specificity of 99% for CTCs, but a sensitivity of 42% has limited their application in predicting GC risk^25^. Genetic alterations and methylation of cfDNA have been detected in early stages of several tumors. A prospective study has indicated that cfDNA methylation may signify the occurrence of GC and 4 other cancers^26^. Nonetheless, concerns have been raised regarding the generally low abundance of cfDNA in early-stage GC and relatively low sensitivity, thus precluding the application of cfDNA alterations as independent biomarkers for GC screening.

**Table 1:** Studies on the performance of PG combined with other biomarkers in predicting GC risk

Reference	Methods	Included biomarkers and factors	Risk groups or score ranges	AUC
Miki 2011^19^	ABC method	*Hp* infection, PG I and PGR (PG I ≤70 ng/mL and PGR ≤3 classified as PG positive)	Group A: *Hp*(−) PG(−) Group B: *Hp*(−) PG(+) Group C: *Hp*(+) PG(+) Group D: *Hp*(−) PG(+)	0.527^†^
Tu 2017^20^	Combination of 5 serum biomarkers	*Hp* infection (score of 7 for *Hp* positive participants) PGR (score of 4 for PGR ≤ 7) PGI (score of 1 for PGI < 30 ng/mL) PGII (score of 0, 1, 3, and 6 for quartiles 1–4, respectively) G-17 (score of 1, 0, 1, and 3 for quartiles 1–4, respectively)	Serum biopsy score, ranging from 0 to 21	0.803*
Cai 2019^21^	Combination of 7 indicators	Age (score of 4, 6 and 9 for ages 50–59, 60–69 and > 69, respectively) Sex (score of 4 for males) Pickled or fried food (score of 2 for regular intake) *Hp* infection (score of 1 for *Hp* positive participants) PGR (score of 3 for PGR < 3.89) G-17 (score of 3 for 1.50–5.70 pmol/L and score of 4 for > 5.70 pmol/L)	0–25, Low risk (≤ 11) Moderate risk (12–16) High risk (17–25)	0.757
Shida 2020^22^	ABC method combined with KK-LC-1	*Hp* infection, PG I, PGR, combining KK-LC-1 expression	ABC method, as in Miki 2011^19^	-
Zeng 2020^23^	Serum miRNA biomarkers combined with PG	miR-101-3p, PG I and PGR	Youden index as cutoff value	0.856

*Hp*, *Helicobacter pylori*; PG, pepsinogen; PGR, the PGI to PGII ratio; G-17, gastrin-17; KK-LC-1, Kita-Kyushu lung cancer antigen-1. ^†^Evaluated by Cai 2019^21^. *Concordance index.

Continually emerging high-throughput multi-omics methods have fostered the development of novel biomarkers. Jin et al.^27^ have constructed a polygenic risk score of 112 single nucleotide polymorphisms, which predicted the risk of incident GC in prospective follow-up; moreover, genetic risk can be partially offset by adhering to a healthful lifestyle. Proteins carry out the functions of genes and determine phenotypes. In a proteomics study involving the full spectrum of gastric lesions in GC progression, our team has reported that altered signatures of APOA1BP, PGC, HPX, and DDT in gastric tissues are associated with the risk of gastric lesion progression and GC occurrence^28^. Integrating these 4 proteomic signatures significantly strengthens the prediction of gastric lesion progression^28^. Two-stage metabolomic profiling of 400 individuals has uncovered several key metabolites, including α-linolenic acid, linoleic acid, and palmitic acid, which may predict the progression of gastric lesions and risk of early GC, thus providing important inputs for defining high-risk populations and detecting GC in early stages^29^. Intriguing findings have also been reported for gastrointestinal microbiota. A recent study has observed enriched Streptococcus anginosus (Sa) and Streptococcus constellatus (Sc) in feces from intraepithelial neoplasia, and early and advanced GCs; the combination of these markers had 91.1% sensitivity in detecting early GC and therefore may provide a promising option for future validation of potential biomarkers^30^.

To date, studies on biomarkers for early detection of GC have been essentially exploratory in nature. Although the use of any single marker is unlikely to have satisfactory performance for GC early detection, much more research is required to develop biomarker panels with sufficient accuracy and sensitivity to precisely predict GC risk by using a combination of biomarkers and other known risk factors^31–33^. Large-scale multicenter prospective studies are warranted, and a thorough evaluation of the affordability and cost-effectiveness after adding biomarkers is needed before the translation of any biomarker combination to general screening practice.

### Determining key elements for implementing refined policies on GC screening

Modification of the current screening strategy requires the implementation of refined health policies for GC screening. To this end, evidence must be gathered regarding screening ages and time intervals for repeated screening. In 2018, the national mass GC screening program in Japan postponed the age for screening initiation to 50 years, without specifying the stopping age^34^. The Korean GC screening guidelines recommend that asymptomatic adults 40–74 years of age undergo gastroscopic screening^35^. The current GC screening programs in China are empirically aimed at benefiting individuals at 40–69 years of age, although the most recent guidelines released by National Cancer Center of China recommend a change in the starting (45 years) and stopping (75 years) ages^36^. In fact, determining key elements for screening guidelines is far beyond an issue of pure science, particularly for national screening programs, which are organized and funded by the government and largely depend on accessible resources. When resources are available, determining the optimal screening starting and stopping ages and appropriate time intervals for repeated screening would require comprehensive evaluation of screening effectiveness and risk prediction for specific population subgroups.

### Encouraging opportunistic screening for improved population coverage in China

Despite all prior accomplishments, population-based, organized GC screening as part of the national key public health programs covers only a portion of eligible individuals in selected high-risk areas. The overall early detection rate of GC remains unfavorable, and substantial disparities remain in achieving standardized diagnosis and treatment in China, a populous developing country. In addition to population-based GC screening programs in both rural and urban China, opportunistic screening mainly by primary medical institutions may be performed on the basis of recommendations made by healthcare providers during routine medical consultations, or self-referral of individuals, thereby serving as a complementary approach to population-based screening for sustainable GC prevention and control in China. Indeed, considering both international experience and domestic circumstances, the opportunistic screening strategy has been successful in encouraging the motivation of local government. In 2021, a total of 2.61 million individuals underwent opportunistic screening for UGI cancers, and 56,677 cases were detected (detection rate of 2.17%).

## Prospects

*China’s Medium-to-Long Term Plan for the Prevention and Treatment of Chronic Diseases (2017–2025)* under the Healthy China Initiative and *Tutorial for Outline of the Healthy China 2030 Plan* issued in 2017 have provided a blueprint for the management of chronic diseases. Because GC is a major public health threat, our mission is to accomplish the strategic goal of GC prevention and control in China. Burgeoning novel experimental and analytical techniques provide new opportunities for biomarker identification and population stratification. Accompanying the flourishing era of precision medicine, we are transiting from a “one-size-fits-all” screening approach to an individualized prevention strategy. Although the road ahead remains long, with multi-center collaborative and multidiscipline-integrated efforts, sustained actions will take us to our destination.
